# Retinal Microvascular Resistance Estimated from Waveform Analysis Is Significantly Higher With a Threshold Value in Central Retinal Vein Occlusion

**DOI:** 10.1167/tvst.9.11.4

**Published:** 2020-12-06

**Authors:** Makiko Matsumoto, Kiyoshi Suzuma, Fumito Akiyama, Kanako Yamada, Shiori Harada, Eiko Tsuiki, Takashi Kitaoka

**Affiliations:** 1 Department of Ophthalmology and Visual Sciences, Graduate School of Biomedical Sciences, Nagasaki University, Nagasaki, Japan; 2 Department of Ophthalmology and Visual Sciences, Graduate School of Biomedical Sciences, Kagawa University, Kagawa, Japan

**Keywords:** central retinal vein occlusion, total capillary resistance, laser speckle flowgraphy, mean blur rate

## Abstract

**Purpose:**

Evaluation of blood flow is useful for understanding the severity of central retinal vein occlusion (CRVO). Actual blood flow may be determined by the resistivity of the retinal vein in CRVO. We have previously evaluated mean blur rate (MBR) to reflect total retinal blood flow velocity in CRVO cases using laser speckle flowgraphy (LSFG). This study evaluated retinal total vascular resistance in CRVO cases using the new index of total capillary resistance (TCR) from LSFG.

**Methods:**

We measured the TCR of 68 CRVO patients who visited Nagasaki University Hospital between 2009 and 2016 and 42 age-matched controls without systemic disease. We compared TCRs among control eyes, CRVO fellow eyes, and CRVO affected eyes. A CRVO threshold value was then obtained from the receiver operating characteristic curve.

**Results:**

MBR was significantly lower for CRVO affected eyes (20.3 ± 8.2) than for control eyes (37.5 ± 8.4; *P* < 0.01) and CRVO fellow eyes (36.4 ± 10.0; *P* < 0.01, Dunn's test). TCR was significantly higher for CRVO affected eyes (1.20 ± 0.55) than for control eyes (0.68 ± 0.2; *P* < 0.01) and CRVO fellow eyes (0.81 ± 0.28; *P* < 0.01, Dunn's test). The threshold for the presence of CRVO was 0.93 and area under the curve was 0.84.

**Conclusions:**

By measuring TCR in addition to MBR, more detailed information regarding CRVO pathology can be obtained.

**Translational Relevance:**

Comparison of values before and after treatment may be useful for evaluating the effects of treatment.

## Introduction

In central retinal vein occlusion (CRVO), the degree of ischemia varies among individual cases and according to the actual stage of the disease.^1^ Evaluation of blood flow is useful for understanding the pathology of diseases such as CRVO, in which dysfunctional blood flow represents the basis of the pathology. Understanding the relationship between actual blood flow and resistivity of the retinal vein is important in CRVO.

Doppler methods for detecting ocular blood flow have long been utilized.^2^^,^^3^ The most well-known approach to detecting the resistivity of blood flow for systemic vascular disease is Doppler waveform analysis. The pulsatility index (PI), one of the parameters representing vascular resistance in an observed region,^4^^,^^5^ is calculated by dividing the peak-to-peak frequency by the mean frequency signal as blood flow.

In a recent study measuring blood flow, optic nerve head (ONH) blood flow (representing blood flow of the whole retina) was measured using laser speckle flowgraphy (LSFG).^6^^–^^11^ LSFG is a recently developed technique that has been used for visualizing blood flow distributions in the ocular fundus. This method is convenient for measuring blood flows and can be useful for clinical applications (e.g., diabetic retinopathy,^12^^–^^18^ glaucoma,^8^^,^^19^^–^^25^ uveitis^26^^–^^28^). We have previously reported LSFG findings in eyes with CRVO.^29^^–^^31^

In this study, we calculated a new parameter similar to the PI by dividing the peak-to-peak frequency by the mean frequency signal as LSFG blood flow. We determined this value as the resistivity of all retinal vessels and refer to it as total capillary resistance (TCR). We compared mean TCRs in patients with CRVO to those in controls and analyzed threshold values using receiver operating characteristic (ROC) curves.

## Methods

This retrospective observational case-control series study was conducted in accordance with the tenets of the Declaration of Helsinki. After approval of the study by the Review Committee of the Institutional Research Board of Nagasaki University Hospital (approval number 19021820-2), CRVO patients were enrolled as the patient group and age-matched volunteers without any systemic diseases were enrolled as the control group. These participants were then evaluated at Nagasaki University Hospital between January 2009 and December 2016. Subjects were excluded if proper measurements could not be obtained (e.g., in patients with cataracts with severe opacity, vitreous hemorrhage, poor mydriasis, or corneal opacity), if the pathology was classified as ischemic-type CRVO by fluorescein angiography, or if the patient showed high myopia. All participants signed a written informed consent form after they were provided with information on the procedures and possible complications.

### LSFG Blood Flow Measurements

Measurements were obtained using a LSFG-NAVI system (Softcare Co., Ltd., Fukuoka, Japan). As previously described,^6^^,^^7^ the LSFG technique can be used to measure ONH blood flow. We evaluated microcirculation at the ONH by measuring the mean blur rate (MBR) of the ONH, as previously reported.^25^^,^^31^^–^^33^ After demarcating a circle around the ONH by hand using an oval band, we then investigated MBRs of the major vessels (arteries and veins) within the area of this circle. Because the MBR in the vessel area includes choroidal blood flow, we subtracted the mean MBR in the tissue area from the mean MBR in the vessel area. Thus, the MBR used to evaluate blood flow in retinal vessels excluded choroidal blood flow.

LSFG can analyze blood flow as a series of pulsatile blood flows over several cardiac cycles for 4 seconds. As a result, LSFG can detect peak-to-peak blood flow in the cardiac cycle. To evaluate peak-to-peak blood flow using LSFG, we calculated beat strength (BS) as being proportional to the amplitude between maximum and minimum blood flow. The formula for calculating BS can be viewed in the patent application W0/2018/003139, Blood Flow Dynamic Imaging Diagnosis Device and Diagnosis Method (https://patentscope.wipo.int/search/en/detail.jsf?docId=WO2018003139).^34^

TCR, the new parameter of resistivity of the retinal vein, is calculated on the ONH for CRVO by the following equation:

TCR=BS,intheareaofONH/MBR

where MBR represents the average blood flow velocity of major vessels (arteries and veins) in the ONH. The parameter BS represents the proportional value of peak-to-peak blood flow corresponding to the major vessels in the ONH. As a result, TCR represents total resistivity throughout all retinal vessels (including the retinal artery, arterioles, capillaries, venules, and central retinal vein).

### Statistical Analysis

The primary objective of this study was to compare TCR values of CRVO patients with those of age-matched controls without systemic diseases and to determine the threshold of TCR. Multiple comparisons of TCR were performed in control eyes, CRVO fellow eyes, and CRVO affected eyes using Dunn's test. The presence or absence of CRVO was determined using the Youden index, with CRVO cases set as true and control cases set as false; the true positives, false positives, true negatives, and false negatives were then calculated to evaluate the use of TCR for this purpose. The accuracy of such judgment was shown as the area under the ROC curve. All statistical analyses were carried out using R 3.5.2 (R Foundation for Statistical Computing, Vienna, Austria). Results are expressed as mean ± standard deviation, unless otherwise indicated. Values of *P* < 0.05 were considered to indicate statistical significance.

## Results

This study assessed a total of 68 eyes in 68 consecutive CRVO patients (40 men, 28 women), with a mean age of 67.8 ± 11.8 years. As age-matched controls, 42 eyes in 42 volunteers without systemic diseases (18 men, 24 women; mean age, 64.9 ± 3.9 years) were assessed. None of the age-matched controls had any past clinical history. Among the total number of CRVO patients, 46 patients had hypertension, 16 patients had diabetes mellitus, one patient had ischemic heart disease, three patients had arrhythmia, three patients had cancer, and 13 patients had no past clinical history. The period from symptom onset to the initial visit to the hospital was 3.6 ± 4.6 months.

**Table 1.** Clinical Characteristics of Each Group

Characteristic	Control Eyes (*n* = 42)	CRVO Eyes (*n* = 68)	*P*
			
Male/female, *n*	18/24	40/28	0.1
Age (y), mean ± SD	64.9 ± 3.9	67.8 ± 11.8	0.06
Duration from CRVO onset to first visit (mo), mean ± SD	—	3.6 ± 5.8	
Past history, *n* (%)			
Hypertension	0	46 (67.6)	0.0
Diabetes mellitus	0	16 (23.5)	0.00004
Ischemic heart disease	0	1 (1.5)	0.43
Arrhythmia	0	3 (4.4)	0.17
Cancer	0	3 (4.4)	0.17

Table 1 summarizes the clinical characteristics of each group. No significant differences were observed between the two groups for sex or age. Table 2 shows the measured values of intraocular pressure (IOP), ocular perfusion pressure (OPP), MBR, and TCR in the control eyes, CRVO fellow eyes, and CRVO affected eyes. No differences in IOP were seen among the three groups (control eyes, 13.4 ± 2.3 mm Hg; CRVO fellow eyes, 14.3 ± 2.6 mm Hg; CRVO affected eyes, 13.7 ± 2.4 mm Hg; not significant). Because many patients in the CRVO group showed hypertension (46 cases, 67.6%), OPP was significantly higher in the CRVO group (CRVO fellow eyes, 55.0 ± 10.4 mm Hg; CRVO affected eyes, 55.7 ± 10.3 mm Hg) than in the control group (49.1 ± 6.8 mm Hg, *P* < 0.01). MBR was significantly lower in CRVO affected eyes (20.3 ± 8.2) than in control eyes (37.5 ± 8.4; *P* < 0.01) and CRVO fellow eyes (36.4 ± 10.0; *P* < 0.01, Dunn's test). TCR was significantly higher in CRVO affected eyes (1.20 ± 0.55) than in control eyes (0.68 ± 0.2; *P* < 0.01) and CRVO fellow eyes (0.81 ± 0.28; *P* < 0.01, Dunn's test).

**Table 2.** Measured Values for Each Group

	Control Eyes (*n* = 42)	CRVO Fellow Eyes (*n* = 68)	CRVO Affected Eyes (*n* = 68)	*P*
IOP (mmHg)	13.4 ± 2.3	14.3 ± 2.6	13.7 ± 2.4	n.s.
OPP^a^ (mmHg)	49.1 ± 6.8	55.0 ± 10.4	55.7 ± 10.3	<0.01
MBR	37.5 ± 8.4	36.4 ± 10.0	20.3 ± 8.2	<0.01
TCR	0.68 ± 0.2	0.81 ± 0.28	1.20 ± 0.55	<0.01

n.s., not significant.

a OPP = 2/3 average artery pressure – IOP.

**Figure 1.**
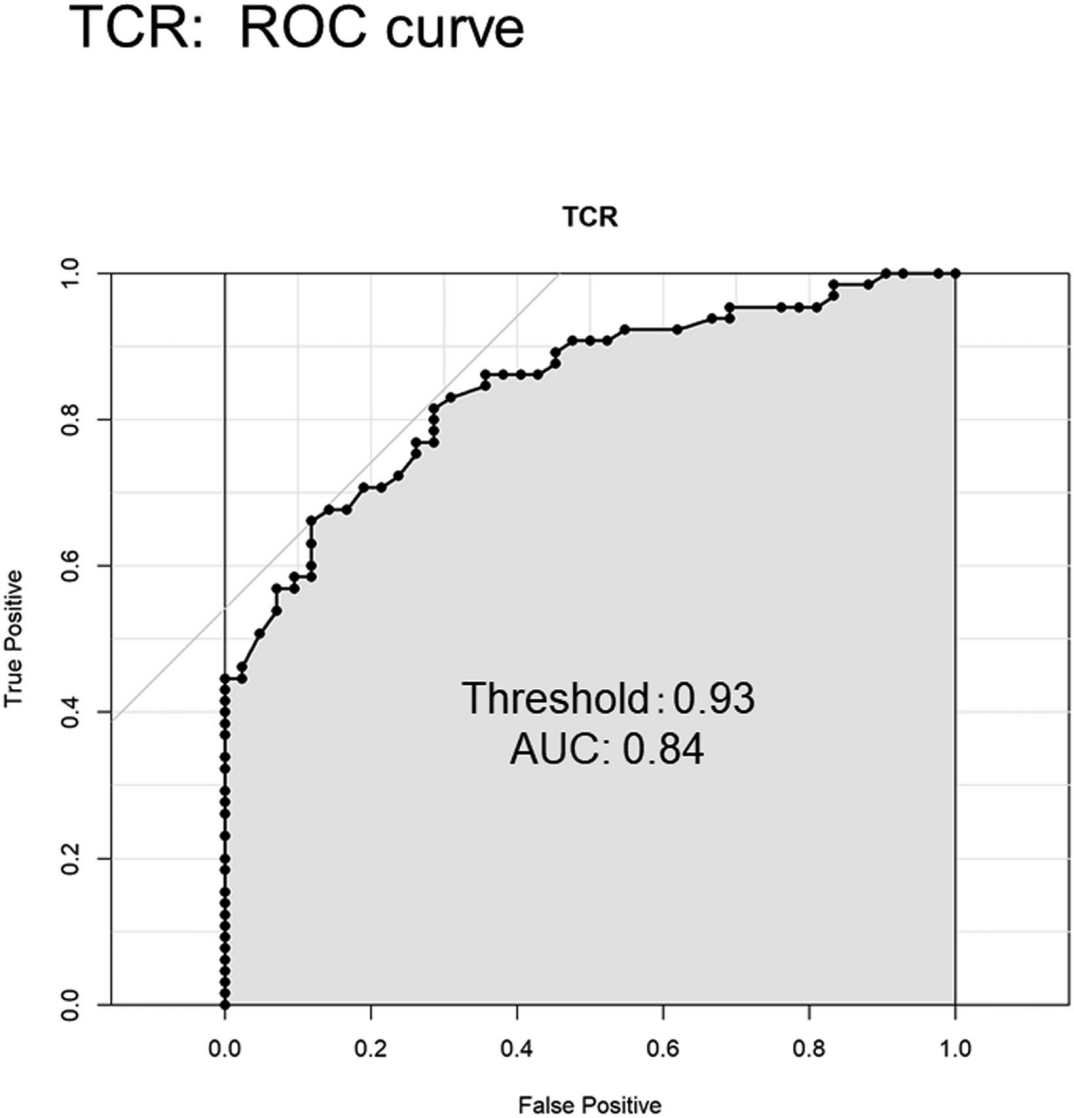
ROC curve for diagnosing the presence or absence of CRVO using TCR. The threshold for the presence of CRVO was 0.93, and the AUC was 0.84.

**Figure 2.**
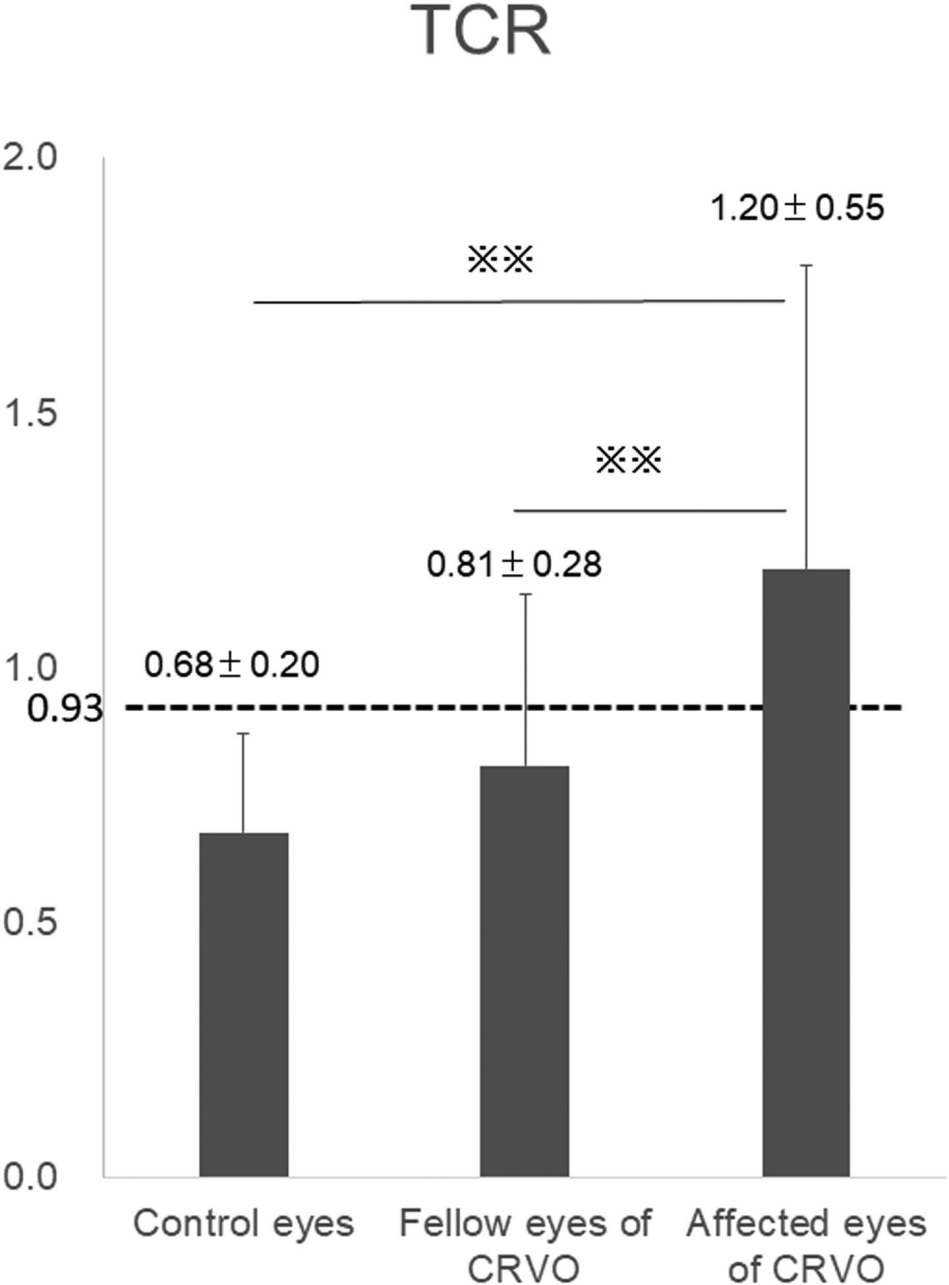
TCR in control eyes, CRVO fellow eyes, and CRVO affected eyes. TCR was significantly higher in CRVO affected eyes than in control eyes or CRVO fellow eyes (^**^P < 0.01 each, Dunn's test). Mean TCR was below the threshold (0.93) in control eyes and CRVO fellow eyes and above the threshold in CRVO affected eyes.

TCR threshold in CRVO eyes was calculated using the Youden index, with CRVO cases set as true and control cases set as false. Figure 1 shows the ROC curve for diagnosing the presence or absence of CRVO using TCR. The threshold for the presence of CRVO was 0.93 and area under the curve (AUC) was 0.84. Figure 2 shows TCR values for the three groups. Mean TCR was below the threshold of 0.93 in control eyes (0.68 ± 0.20) and CRVO fellow eyes (0.81 ± 0.28) and was above the threshold in eyes with CRVO (1.20 ± 0.55).

## Discussion

The prevalence of CRVO is reportedly strongly associated with aging.^35^ Mean age in our CRVO cases was 67.8 ± 11.8 years, not significantly different from previous reports.^36^^–^^38^ Hypertension is the most representative risk factor for CRVO.^36^^,^^39^^–^^41^ In our cases, 46 of 68 patients (67.6%) showed hypertension, lower than previously reported (89.2%).^37^ Diabetes and ischemic heart disease are also known to be risk factors for CRVO.^37^^,^^42^^,^^43^ In our study, 16 patients (23.5%) had diabetes and one patient (1.5%) had ischemic heart disease.

OPP was significantly higher in the CRVO cases than in the controls because of the influence of blood pressure (OPP = 2/3 average artery pressure – IOP). The MBRs of affected eyes in CRVO were significantly lower than those of both fellow eyes and control eyes. The TCR of affected eyes in CRVO was significantly higher than that of both fellow eyes and control eyes. When CRVO develops, TCR increases and MBR decreases.

Comparison of CRVO affected eyes with CRVO fellow eyes is also important; however, the blood flow in fellow eyes was originally low,^44^ and some patients have CRVO in both eyes,^45^ so we consider fellow eyes to differ from normal eyes. In this study as well, OPP and TCR were higher in CRVO fellow eyes than in controls; therefore, comparison with normal eyes appears desirable to obtain threshold values.

This study was able to clarify the threshold for the presence or absence of CRVO in TCR. The threshold for the presence of CRVO was 0.93. The AUC of 0.84 suggests that TCR could serve as a good indicator of CRVO. We have previously reported that MBR increased after intravitreal injection of anti-vascular endothelial growth factor (VEGF) in macular edema with CRVO.^29^^,^^31^ We also reported that patients with higher MBR after anti-VEGF treatment displayed a better prognosis, and the prognosis was poor in cases where MBR did not rise after anti-VEGF treatment. LSFG is a non-invasive clinical test with a shorter recording time, and the results can be evaluated over time at each visit. Although the degree of ischemia in CRVO can be observed by fluorescein angiography, conducting an examination every visit is not practical, because this method involves risks such as shock and is relatively time consuming. CRVO can cause sudden ischemia. If the MBR decreases and TCR increases during follow-up, careful consideration of the next treatment is warranted.

CRVO studies have suggested that a blockage of veins causes elevated resistivity in retinal vessels, as well as an increased TCR, as the resistivity of the ONH rises. TCR thus offers a very useful parameter for quantifying vessel blockage. Assessment of TCR in addition to MBR can be expected to facilitate obtaining a more detailed understanding of the pathophysiology of CRVO and would appear to offer potential in evaluating CRVO treatment methods.

This study carefully excluded ischemic CRVO due to the small number of cases and the possibility of different pathologies. The limitations of this study were the small number of cases (68 CRVO, 42 controls), the exclusion of ischemic CRVO, the fact that participants were all Asian, and the single-center design of the study. Further studies are needed to accumulate a greater number of cases and evaluate ischemic CRVO (as ischemic type alone or including ischemic type) using LSFG.
